# Efficacy of *Andrographis paniculata* against extended spectrum β-lactamase (ESBL) producing *E. coli*

**DOI:** 10.1186/s12906-018-2312-8

**Published:** 2018-09-03

**Authors:** Ubaid Rasool, Priya S, Afsana Parveen, Saroj Kumar Sah, Hemalatha S

**Affiliations:** School of Life Sciences, B.S. Abdur Rahman Crescent Institute of Science and Technology, Vandalur, Chennai, 600048 India

**Keywords:** *A. paniculata*, ESBL, Antibacterial, Molecular docking, Gene expression

## Abstract

**Background:**

*A. paniculata* is widely known for its medicinal values and is traditionally used to treat a wide range of diseases such as cancer, diabetes, skin infections, influenza, diarrhoea, etc. The phytochemical constituents of this plant possess unique and interesting biological activities. The main focus of this study was to evaluate the antibacterial property of crude ethyl acetate (CEA) extract of *A. paniculata* against *E. coli* clinical isolates along with molecular docking of 10 different bioactive components from this plant with CTX-M-15.

**Methods:**

CEA extract was subjected to phytochemical and FTIR analysis. The *E. coli* isolates were tested for antibiotic susceptibility through disk-diffusion method to observe their resistance pattern towards different antibiotics. Antibacterial activity and biofilm assay were performed through broth microdilution using a 96-well microplate. CEA extract was further utilized to observe its effect on the expression of a gene encoding CTX-M-15. Finally, *in-silico* studies were performed where 10 different bioactive compounds from *A. paniculata* were molecularly docked with CTX-M-15.

**Results:**

Phytochemical and FTIR analysis detected the presence of various secondary metabolites and functional groups in CEA extract respectively. Molecular docking provided the number of residues and bond lengths together with a positive docking score. Antibiotic susceptibility showed the multi-drug resistance of all the clinical strains of *E. coli*. The antibacterial and antibiofilm efficiency of CEA extract (25, 50 and 100 μg/ml) was tested and 100 μg/ml of the extract was more effective in all the strains of *E. coli*. All 3 ESBL producing strains of *E. coli* were subjected to gene expression analysis through PCR. Strains treated with 100 μg/ml of the extract showed a downregulation of the gene encoding CTX-M-15 compared to untreated controls.

**Conclusions:**

The utilization of CEA extract of *A. paniculata* proved an economical way of controlling the growth and biofilm formation of ESBL strains of *E. coli*. CEA extract was also able to downregulate the expression of a gene encoding CTX-M-15. Molecular docking of 10 different bioactive compounds from *A. paniculata* with CTX-M-15 provided the residues and bond lengths with a positive docking score.

**Electronic supplementary material:**

The online version of this article (10.1186/s12906-018-2312-8) contains supplementary material, which is available to authorized users.

## Background

*Andrographis paniculata* is widely used for medicinal purposes [1, 2]. The primary component of this plant that possesses medicinal values is andrographolide, a diterpene lactone which has been reported by various researchers as anti-cancerous [3], anti-HIV [4], cardioprotective [5], hepatoprotective [6] apart from other medically important values. The other active components that are present in *A. paniculata* include andrographolide D, homoandrographolide, andrographosterin and stigmasterol [7]. Zaiden and co-workers reported the antimicrobial effect of water extracts of *A. paniculata* against both Gram-positive and Gram-negative bacteria [8]. Bobbarala and co-workers reported higher antibacterial activity of methanolic extracts of *A. paniculata* (95% inhibition of test organisms) compared to chloroform (80% inhibition of test organisms) and hexane (65% inhibition of test organisms) extracts [9].

The main motive behind this study was to study the effect of CEA extract of *A. paniculata* on the growth and biofilm formation in 3 ESBL producing strains and one ATCC strain of *E. coli* together with *in-silico* studies. *E. coli* strains were also subjected to DNA isolation and gene expression analysis through polymerase chain reaction (PCR) to study the effect of CEA extract on the expression of a gene encoding CTX-M-15.

## Methods

### Bacterial strains and *A. paniculata* leaves

ESBL strains were collected from Tagore Medical College, Chennai, India after a proper approval from the institutional ethics committee. ATCC strain of *E. coli* was provided from the departmental stock at School of Life Sciences, BSACIST. All the strains were properly sub-cultured and maintained as glycerol stocks and slants. The *Andrographis paniculata* leaves were collected from BSACIST, Chennai. The samples were identified and authenticated by Dr. D. Narasimhan, Botanist, Madras Christian College, Chennai. A specimen of the plant was deposited at School of Life Sciences, BSACIST, Chennai (Accession number: SLS-BSAU-16100).

### Sample preparation, solvent extraction and phytochemical analysis

Leaves of *A. paniculata* were collected and dried in hot air oven at 60 °C and were crushed to powder. 50 g of the powder was soaked in 250 ml of ethyl acetate and incubated in a shaker for 48 h followed by filtration through Whatman filter paper. The extracted residue was air dried and a stock concentration of 1 mg/ml was prepared. The CEA extract was qualitatively tested for the presence of tannins, flavonoids, carbohydrates, terpenoids, saponins and amino acids [10–12].

### FTIR analysis

CEA extract of *A. paniculata* was subjected to FTIR analysis to get a knowledge about the functional groups present. FTIR analysis is an established tool that is very helpful in characterizing and identifying various functional groups that may be available in any unidentified plant extract [13, 14]. Both liquids, as well as solid samples, can be used to carry out this analysis [15].

### In-silico studies

Molecular docking of 10 different active components of *A. paniculata* was carried out with CTX-M-15. SYBYL®-X 1.3 (http://www.tripos.com) was the software package used to carry out the calculations. This software package runs on 32 or 64-bit operating systems (Windows XP SP3 and Windows 7).

### Ligand preparation

A total of 38 bioactive compounds from *A. paniculata* were selected, out of which 10 bioactive compounds were finally chosen for molecular docking studies. The three-dimensional structures of all the bioactive compounds used were retrieved from PubChem and the final optimized ligands were used for molecular docking.

### Protein structure preparation

The crystal structure of bacterial target protein CTX-M-15 (PBD ID: 5 T66) was retrieved from the PDB database (http://www.rcsb.org) [16]. Receptor protein was made free from all the crystallographic substructures and water molecules. This was followed by the addition of necessary hydrogen atoms and charges along with Gasteiger-Marsili. Trivial portable operating system (TRIPOS) was employed for the minimization process and protein ProtoMol was automatically generated. The final structure was viewed using PyMOL (http://www.pymol.org).

### Molecular docking

Molecular docking is a tool that helps in predicting the orientation of one molecule to a second when they are bound to each other forming a stable complex. That orientation, in turn, may be used for the prediction of binding affinity or the strength with which the two molecules are associated. Docking finds an important role in rational drug designing through the prediction of orientation between drug molecules and their protein targets [17]. In this particular study, a total of 10 compounds were docked with CTX-M-15 protein to get the binding affinities, hydrogen bonds and hydrophobic interactions.

### Antibiotic susceptibility testing and phenotypic ESBL detection

Susceptibility testing of clinical isolates and an ATCC strain of *E. coli* was evaluated via disk diffusion method as described by Ekwealor et al. (2016) with some slight modifications [18]. LBA plates were prepared and seeded with an overnight culture of all three clinical isolates and an ATCC strain of *E. coli*. Following antibiotic discs were used for susceptibility testing: Amoxicillin (25 μg/disc), Amoxicillin and clavulanic acid (20/10 μg/disc), Cefotetan (30 μg/disc), Aztreonam (30 μg/disc), Ceftazidime (30 μg/disc), Ceftriaxone (30 μg/disc), Cefazolin (30 μg/disc). A single disc of each antibiotic was placed on the plates seeded with the overnight cultures of the bacterial isolates. Phenotypic ESBL detection was carried out through combined disc diffusion test following clinical laboratory standard Institute (CLSI) guidelines [19]. Each plate seeded with a clinical isolate of *E. coli* was subjected to phenotypic ESBL confirmatory test by placing two antibiotic discs of ceftazidime and ceftazidime + clavulanic acid. A 5 mm increase in the zone of CAZ + CA compared to CA was considered as ESBL positive. All susceptibility as well as ESBL confirmatory test plates were incubated at 37 °C for 24 h and were observed for the zone of inhibition.

### Multiple antibiotic resistance indices (MARI)

MARI calculations for all the clinical isolates and an ATCC strain of *E. coli* were carried out by dividing the number of antibiotics to which the bacteria showed resistance by the total number of antibiotics to which the bacteria were subjected [18]. MARI calculations were carried out for all three clinical isolates and an ATCC strain of *E. coli*.

### Antibacterial activity through broth microdilution method

In vitro antibacterial activity of CEA extract against three ESBL producing and one ATCC strain of *E. coli* was performed through the modified broth microdilution method [20] using a 96-well microplate. Three different dilutions of CEA extract (25, 50 and 100 μg) were prepared with LB broth and overnight cultures of all *E. coli* strains, adjusted to 0.5 McFarland standard, were added to the prepared dilutions. Positive and negative controls were properly used. The plate was incubated at 37 °C for 12–24 h and the growth of all *E. coli* strains was observed as turbidity using a microplate reader (EnSpireTM Multilabel Reader 2300, S.No. 2300096) at 600 nm. The obtained values were graphically plotted. This experiment was performed in triplicates.

### Biofilm assay using a 96-well microplate

In vitro biofilm inhibition potential of CEA extract against three ESBL and one ATCC strains of *E. coli* was performed as described [21]. Three different dilutions of CEA extract (25, 50 and 100 μg) were prepared with LB broth and all *E. coli* strains, adjusted to 0.5 McFarland standard, were added to the prepared dilutions. Positive and negative controls were properly used. The plate was incubated at 37 °C and 200 rpm in an incubator shaker for 12–48 h. The cells were later discarded and the plate was washed with distilled water in order to remove the unattached cells. 0.1% of crystal violet stain was added to each well and the plate was incubated at room temperature for 10 min. Excess stain was removed by washing so as to decrease the background staining. The plate was kept for drying overnight and 30% glacial acetic acid was added to each well. Spectrophotometric measurements were recorded at 517 nm and the obtained values were graphically plotted. This experiment was performed in triplicates.

### DNA isolation and gene expression analysis by polymerase chain reaction (PCR)

Three ESBL strains of *E. coli* were subjected to DNA isolation and gene expression analysis through PCR. All strains were treated with 100 μg/ml of CEA extract of *A. paniculata* and incubated at 37 °C for 24 h. Strains without treatment served as controls. After proper incubation, 1 ml of solution from each tube was transferred to 1.5 ml Eppendorf tubes and centrifuged at 12000 rpm for 5 min. The supernatant was discarded and the pellet was washed with distilled water. Finally, 250 μl of autoclaved distilled water was added to the pellet, tubes were placed in a thermostat at 100 °C for 10 min (heat lysis) and then centrifuged at 12000 rpm. The supernatant was transferred to another tube and stored at − 80 °C for Agarose gel electrophoresis [22].

DNA samples from all the isolates were amplified with bla_CTX-M-15_ gene. The amplification was carried out on a Mastercycler nexus gradient (Eppendorf, USA). The reaction mixtures comprised of 5 μl of 2× Redeye Master Mix (Amplicon III), 2 μl of 1 M CTX-M-15 forward (CACGTCAATGGGACGATGT) and reverse (GAAAGGCAATACCACCGGT) primer each and 3.0 μl of template DNA. The final reaction volume was 10 μl. The amplification reaction was carried out as the follows: Initial denaturation at 94 °C for 5 min, accompanied by 35 cycles of denaturation at 95 °C for the 30 s, annealing at 55 °C and extension at 70 °C for 1 min, and the final extension at 72 °C for 10 min. The PCR product was analyzed by electrophoresis in 1.5% *w*/*v* agarose gel at 60 V for 60 min. 1× Tris-acetate- EDTA buffer (1X TAE buffer), pH -7.6, 20 mM acetic acid, 1 mM EDTA) and 100 bp DNA ladder (Gene Direx) was used. The gel was stained using ethidium bromide and imaged under ChemiDoc MP System (Bio-Rad, USA 2013) after 1 h of electrophoresis.

## Results

### Phytochemical and FTIR analysis

The CEA extract of *A. paniculata* was subjected to qualitative phytochemical analysis and it was observed that the extract tested positive for terpenoids and saponins whereas the extract tested negative for tannins, flavonoids, carbohydrates and amino acids. FTIR analysis of *A. paniculata* extract was carried out to identify the functional groups present in the extract. The wavenumbers and their corresponding functional groups are presented in Table 1. An Additional file 1 gives a diagrammatic representation of the FTIR spectrum obtained.

**Table 1:** Wave number and the corresponding possible functional groups present in the extract

S No	Wavenumber (cm^− 1^)	Functional groups
1	413.656	Bromide. Iodide
2	607.467	Chloroalkanes
3	1037.52	Amines
4	1241.93	Alkyl Halide
5	1373.07	Alkane
6	1447.31	Alkane or Organophosphorus
7	1734.66	Carbonyl
8	2860.88	Aldehyde
9	2927.41	Alkanes and Alkyls

### In-silico studies

**Fig. 1:**
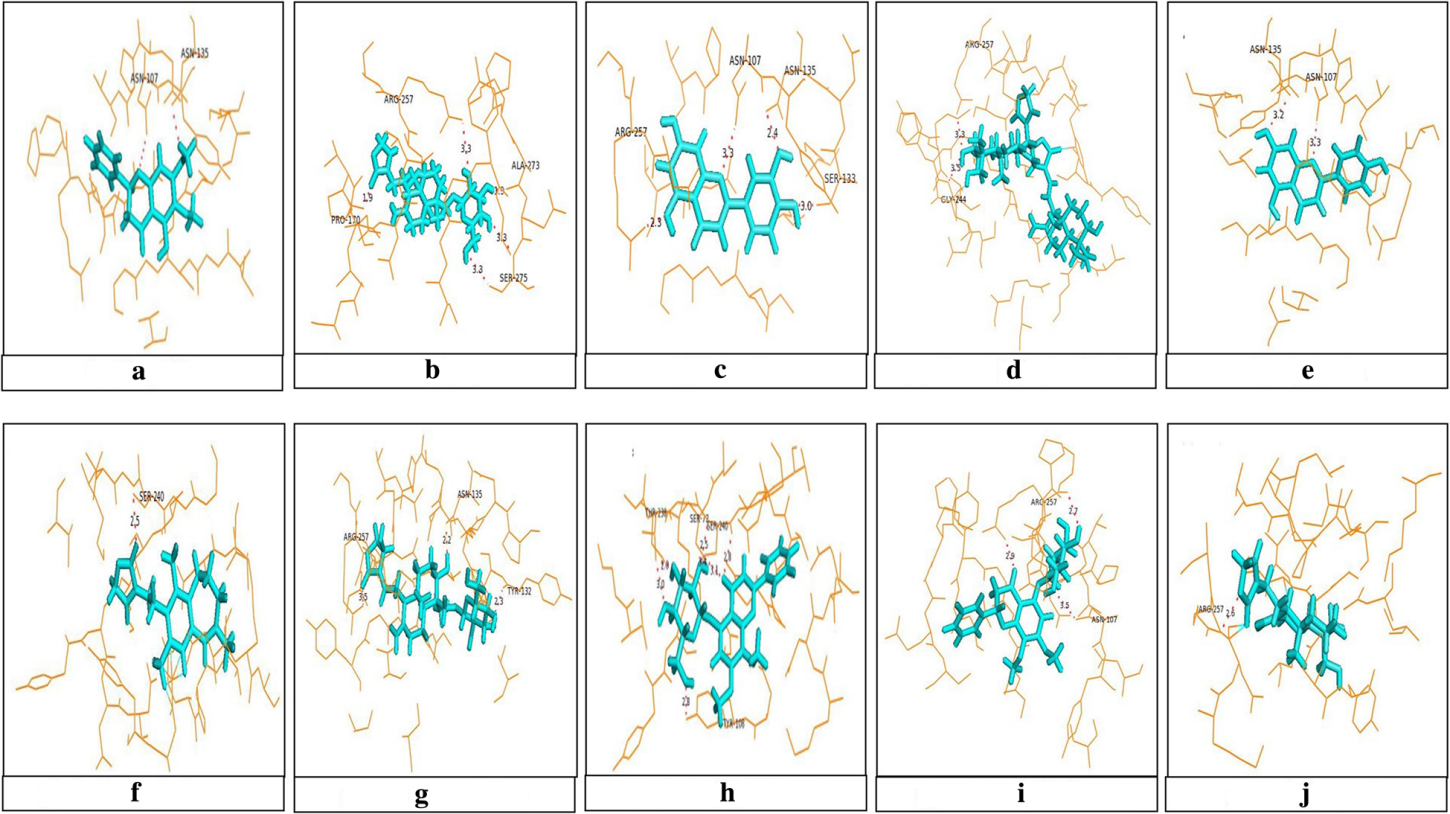
Molecular docking of CTX-M-15 with 10 different bioactive compounds from *A. paniculata.* **a** Onysilin, **b** Neoandrographolide, **c** Luteolin, **d** Bisandrographolide A, **e** Apigenin, **f** Andrographolactone, **g** Andrographiside, **h** Andrographidine C, **i** Andrographidine A, **j** Andrograpanin

The three-dimensional structures of all 10 bioactive compounds were retrieved from PubChem and the final optimized ligands were used for molecular docking. Docking analysis of CTX-M-15 with the 10 bioactive compounds along with the residues and the bond length of each compound is shown in Fig. 1**.** An Additional file 2 gives tabulated information about the residues and bond lengths of each compound.

### Antibiotic susceptibility testing and phenotypic ESBL detection

From susceptibility testing, it was observed that the clinical isolates of *E. coli* were resistant to most and sensitive to a few antibiotics. MARI calculations were also calculated which give an idea about the misuse of antibiotics leading to resistance. MARI calculations were calculated by dividing the number of antibiotics towards which the isolates showed resistance by the total number of antibiotics to which the isolates were subjected. Susceptibility results and MARI calculations are presented in Table 2. Furthermore, the phenotypic detection of ESBL was carried out by the double disc diffusion method. An Additional file 1 provides the diagrammatic representation of antibiotic susceptibility results and the phenotypic detection of ESBL production.

**Table 2:** Susceptibility details of *E. coli* isolates towards different antibiotics

Strain	Source of isolates	Resistance details	Sensitive	MARI calculations
*E. coli* strain 1	Urine	CAZ, AMX, AMC, CTN, AT, VA,	CTR, CZ	0.75
*E. coli* strain 2	Urine	CAZ, AMX, AMC, CTR, CTN, AT, VA, CZ		1
*E. coli* strain 3	Urine	CAZ, AMX, AMC, CTR, CTN, AT, VA, CZ		1

*AMX* Amoxicillin (25 mcg/disc), *AMC* Amoxicillin and clavulanic acid (20/10 mcg/disc), *CTN* Cefotetan (30 mcg/disc), *AT* Aztreonam (30 mcg/disc), *CAZ* Ceftazidime (30 mcg/disc), *CTR* Ceftriaxone (30 mcg/disc), *CZ* Cefazolin (30 mcg/disc)

### Antibacterial activity through broth microdilution

**Fig. 2:**
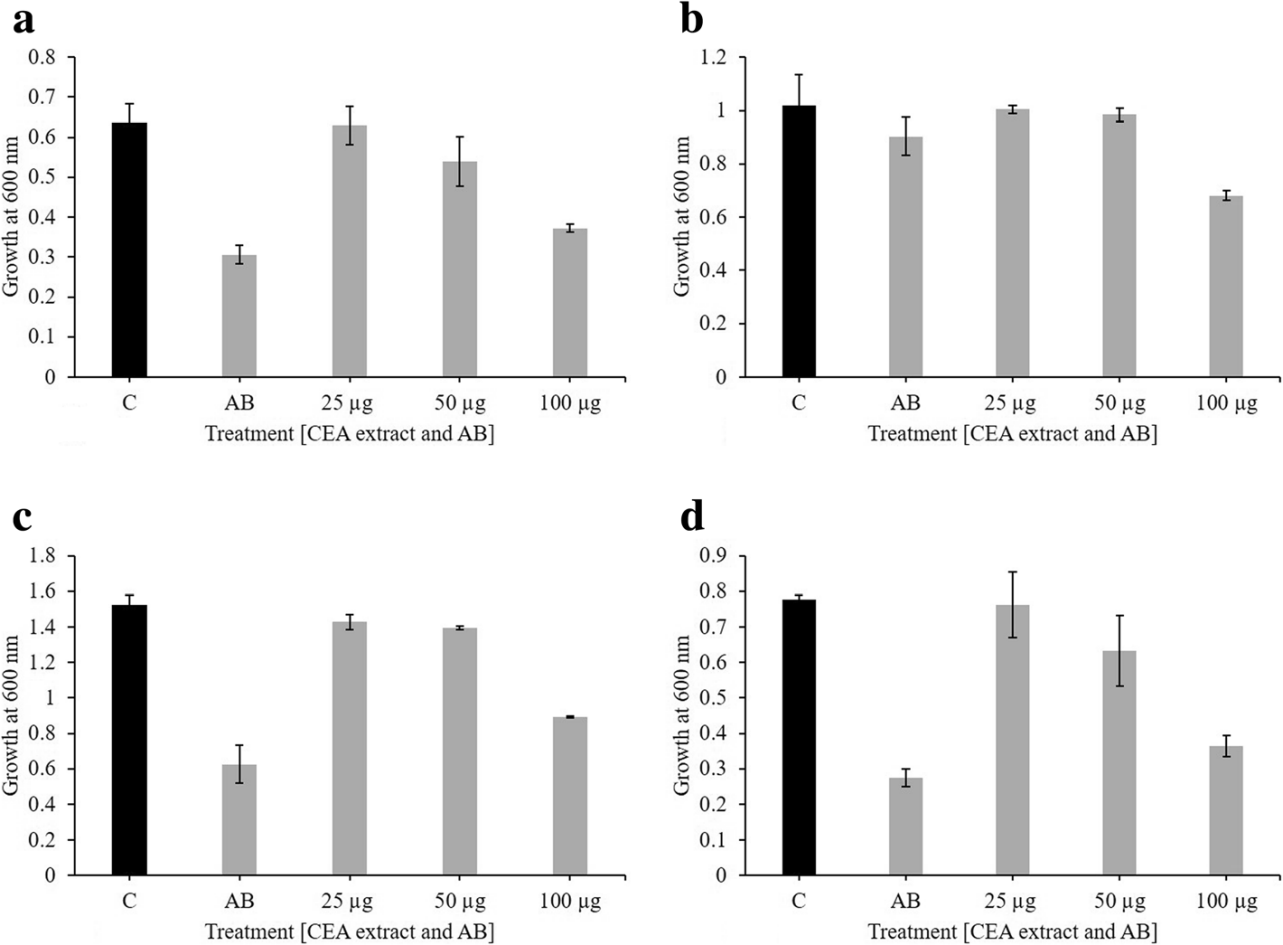
Antibacterial activity of CEA extract through broth microdilution method. The picture shows the effect of 25, 50 and 100 μg/ml of CEA extract on the growth of (**a**) *E. coli* strain 1 (**b**) *E. coli* strain 2 (**c**) *E. coli* strain 3 and (**d**) ATCC strain of *E. coli*

Three different concentrations of the extract (25, 50 and 100 μg/ml) used to check the antibacterial effect provided promising results. 100 μg/ml of extract was most effective in controlling the growth of all *E. coli* strains and the effect was almost as good as the antibiotic. For *E. coli* strain 2, the extract was more effective compared to the antibiotic. 25 μg/ml of the extract was least effective and the growth of *E. coli* strains was almost similar to that of the untreated strains (Fig. 2).

### Biofilm inhibition

**Fig. 3:**
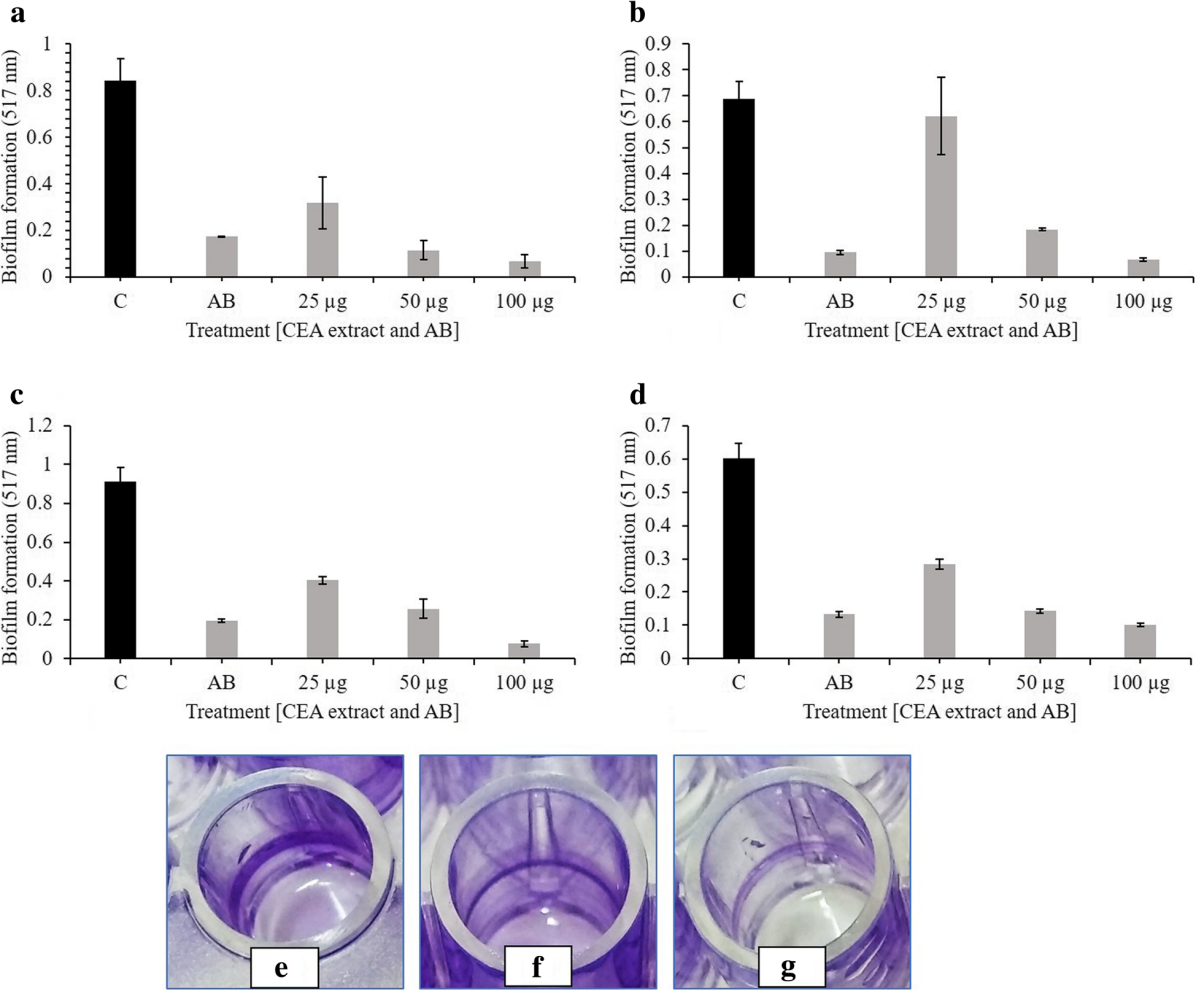
Biofilm inhibition by CEA extract. The picture displays the effect of 25, 50 and 100 μg/ml of CEA extract on the biofilm formation of (**a**) *E. coli* strain 1 (**b**) *E. coli* strain 2 (**c**) *E. coli* strain 3 and (**d**) ATCC strain of *E. coli*. (**e**, **f**, **g**) The formation of different concentrations of biofilm around the walls in a 96-well microplate

Different concentrations of the extract used to check the biofilm inhibition potential against all the *E. coli* strains proved effective. 100 μg/ml of the extract was more effective than the antibiotic in inhibiting the biofilm formation whereas, 50 μg/ml of the extract and the antibiotic had a comparable effect on the biofilm inhibition in all the *E. coli* strains. 25 μg/ml of the extract was least effective (Fig. 3).

### DNA isolation and gene expression analysis by PCR

**Fig. 4:**
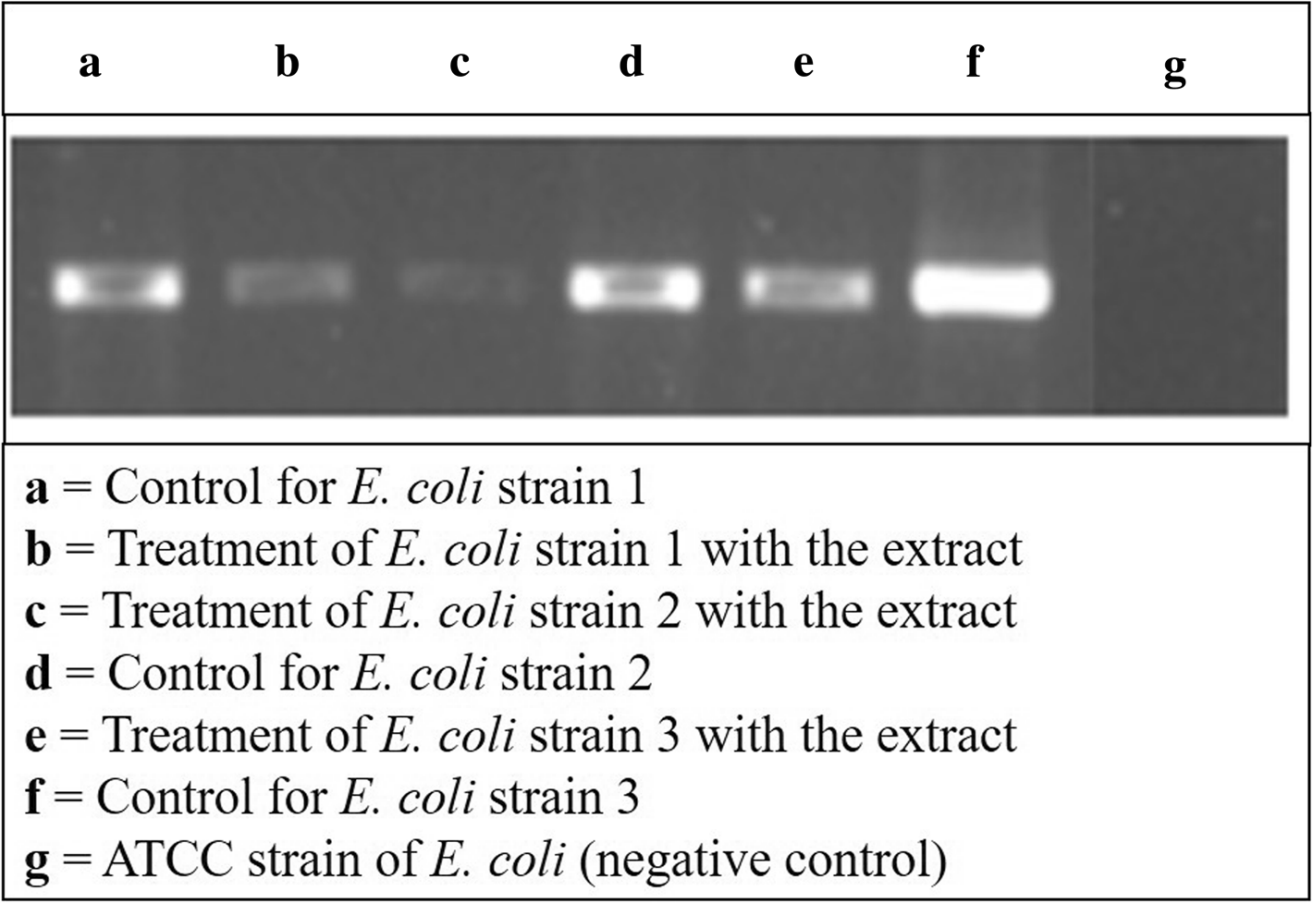
Gene expression analysis through PCR. PCR amplification of gene encoding CTX-M-15 (410 bp) in control and treated *E. coli* strains. (**a**) control with strain 1; (**b**) CEA extract treatment with strain1; (**c**) treatment with strain 2; (**d**) control with strain 2; (**e**) treatment with strain 3; (**f**) control with strain 3; (**g**) ATCC (negative control). The figure gives a description about the CTX-M-15 gene expression with and without the treatment of ethyl acetate extract

The genomic DNA of ESBL producing strains of *E. coli* was isolated and amplified with bla_CTX-M-15_ gene to analyse the gene expression before and after treatment with CEA extract of *A. paniculata*. The results that were obtained after amplification displayed the downregulation of bla_CTX-M-15_ gene in treated strains 2 and 3 when compared to control. The intensity of bands (high, low and medium) was analyzed which corresponded to different gene expressions. ATCC strain of *E. coli* which was used as a negative control showed no expression at all (Fig. 4).

## Discussion

*A. paniculata* has a long history of being utilized as an oriental and Ayurvedic medicine. Genus *Andrographis* belongs to the family Acanthaceae which includes around 40 species. *A. paniculata* is commonly known as the King of Bitters and is native to peninsular India and Sri Lanka. This plant also occupies different regions of Southeast Asia, China, America, West Indies and Christmas Island. The wide distribution of *A. paniculata* accounts for its medicinal values and its growth in most of the soil types. The aerial parts, as well as the roots of *A. paniculata*, have served the purpose of traditional medicine in countries like India, China, Thailand and some Southeast Asian countries. Extensive studies have been conducted by researchers across the world and particularly in Asia owing to the medicinal properties of *A. paniculata*. Phytochemical analysis of *A. paniculata* has unveiled a wide range of compounds such as labdane diterpenoid lactones, flavonoids and miscellaneous compounds. *A. paniculata* has also been reported to display a wide range of pharmacological properties [23]. The antibacterial properties of *A. paniculata* extracts have been reported in some previously carried out studies with different bacterial strains. Leelarasamee and co-workers reported a significant antimicrobial activity of crude extract of *A. paniculata* against *Salmonella, Shigella, Escherichia coli*, *Streptococci,* and *Staphylococcus aureus* at a concentration of 25 mg/ml [24]. An investigation conducted by Mishra and co-workers reported the growth inhibitory effect of ethanol extracts of aerial parts of *A. paniculata* against *E. coli* along with other Gram-positive and Gram-negative bacteria [25]. The present study focussed on utilizing the lesser concentration of CEA extract from *A. paniculata* where 100 μg/ml of the extract proved efficient in controlling the growth and biofilm formation in three different clinical strains and one ATCC strain of *E. coli*. 100 μg/ml of CEA extract showed comparable effects as that of the antibiotic in inhibiting the growth of *E. coli* strains and moreover, CEA was more effective in inhibiting the biofilm of *E. coli* strains compared to the antibiotic. A study conducted by Sule and co-workers reported the antibacterial activity of three different extracts (dichloromethane, methanol, and aqueous) of *A. paniculata* against 12 skin infection causing pathogenic bacterial strains. The extracts showed significant effects against all the tested bacterial strains at a concentration of 1000, 500, and 250 μg/disc [26]. Apart from the antibacterial and antibiofilm activity of CEA extract, molecular docking of 10 different bioactive compounds from *A. paniculata* with CTX-M-15 protein revealed a positive docking score. From the docking results, it can be hypothesized that bioactive compounds from *A. paniculata* may be a better source for inhibiting the CTX-M protein to control the ESBL producing *E. coli* strains. CTX-M beta-lactamases are a growing family of enzymes which are characterized by a selective hydrolysis of ceftriaxone and cefotaxime and more specifically ceftazidime. CTX-M-15 type ESBLs were first reported in *K. pneumoniae*, *E. coli* and human isolates of *Enterobacter aerogenes* from India and Japan. Since that time, CTX-M-15 type ESBLs have been recognized in Enterobacteriaceae strains from various countries like the United Kingdom, Bulgaria, Canada, Russia, Poland, Turkey and France. Extensive research has been conducted on CTX-M-15 type ESBLs in industrialized countries where *E. coli* and Klebsiella spp. are the most common cause of urinary tract infections [27]. In the present study, the molecular docking reports on CTX-M-15 and the bioactive compounds from *A. paniculata* are the first to be reported.

ESBL production by *Enterobacteriaceae* strains has been a cause of resistance against various antimicrobial agents which in turn presents a hindrance in clinical practice making it difficult to treat infections [28]. The resistance mechanisms that ESBL strains have developed need to be keenly observed so that novel and effective antimicrobial agents can be discovered and designed properly [29]. In the present study, the variable expression of a gene encoding CTX-M-15 was observed when treated with CEA extract of *A. paniculata* in 3 different ESBL producing strains of *E. coli*. The study presented an overview of the CEA extract of *A. paniculata* and its strong ability to inhibit the production of ESBL at the molecular level which may act as an alternative to fight the bacterial resistance.

The overall significance of the present study lies in the fact that ESBL producing strains are developing at a fast pace which in turn increases the percentage of antibiotic resistance. In order to stop this increasing menace of antibiotic resistance, a continuous research to find more novel antibacterial agents needs to be carried out. In this study, we presented an approach towards finding a possible way to stop the ESBL producing clinical strains of *E. coli* and since CTX-M-15 type ESBLs are more prevalent in India and more specifically the southern part, hence the strains positive for CTX-M-15 were chosen for this study. Moreover, the literature about *A. paniculata* which gives an idea about the overwhelming applications of this very plant in the medicinal field made us choose this plant for the present study.

## Conclusions

This study proved an effective and an economic way of controlling the growth and biofilm formation in clinical isolates of *E. coli*. Phytochemical and FTIR analysis helped to know about the presence of metabolites and functional groups present in the extract. Susceptibility testing presented the resistance patterns of *E. coli* clinical isolates towards different antibiotic formulations. Antibacterial and antibiofilm activity at lesser concentrations of CEA extract proved effective. CEA extract was also able to downregulate the expression of a gene encoding CTX-M-15. Finally, *in-silico* studies of 10 different bioactive compounds from *A. paniculata* with CTX-M-15 provided the residues and bong lengths with a positive docking score.

### Limitations

- The antibacterial activity of CEA extract can extend to a wide range of other drug-resistant bacteria
- Molecular docking can extend to other bioactive compounds
- Different extracts of *A. paniculata* can be prepared and evaluated further
- Biofilm inhibition assay can extend to more bacterial isolates

## Additional files

Additional file 1: FTIR analysis, antibiotic susceptibility testing and phenotypic detection of ESBL production. The data in the file includes the FTIR spectra of CEA extract, antibiotic susceptibility data of three clinical strains of *E. coli* and double disk diffusion test for ESBL production. (PDF 180 kb)

Additional file 2: Residues and bond lengths of 10 bioactive compounds of *A. paniculata* docked with CTX-M-15. The data in the file includes the tabular representation of the docking results showing the residues and bond lengths of 10 bioactive compounds of *A. paniculata* with CTX-M-15. (PDF 15 kb)

## Abbreviations

ALA: Alanine
AMC: Amoxicillin and clavulanic acid (20/10 mcg/disc)
AMX: Amoxicillin (25 mcg/disc)
ARG: Arginine
ASN: Asparagine
AT: Aztreonam
ATCC: American type culture collection
CAZ: Ceftazidime
CEA: crude ethyl acetate
CLSI: Clinical Laboratory Standard Institute
CTN: Cefotetan
CTR: Ceftriaxone
CTX-M-15: active on CefoTaXime
CZ: Cefazolin
DNA: Deoxyribonucleic acid
ESBL: extended spectrum β-lactamase
FTIR: Fourier transform infrared spectroscopy
GLY: Glycine
HIV: Human immunodeficiency virus
LBA: Luria-Bertani agar
MARI: multiple antibiotic resistance indices
PCR: polymerase chain reaction
PDB: protein databank
PRO: Proline
SER: Serine
THR: Threonine
TYR: Tyrosine
