# Supplementary material for: Efficacy of Andrographis paniculata against extended spectrum β-lactamase (ESBL) producing E. coli
Source: BMC Complement Altern Med. 2018 Sep 3;18:244. doi: 10.1186/s12906-018-2312-8 (PMC6122548; doi:10.1186/s12906-018-2312-8)
Supplement: Supplementary file 1 — FTIR analysis, antibiotic susceptibility testing and phenotypic detection of ESBL production. The data in the file includes the FTIR spectra of CEA extract, antibiotic susceptibility data of three clinical strains of E. coli and double disk diffusion test for ESBL production. (PDF 180 kb) [file 12906_2018_2312_MOESM1_ESM.pdf]

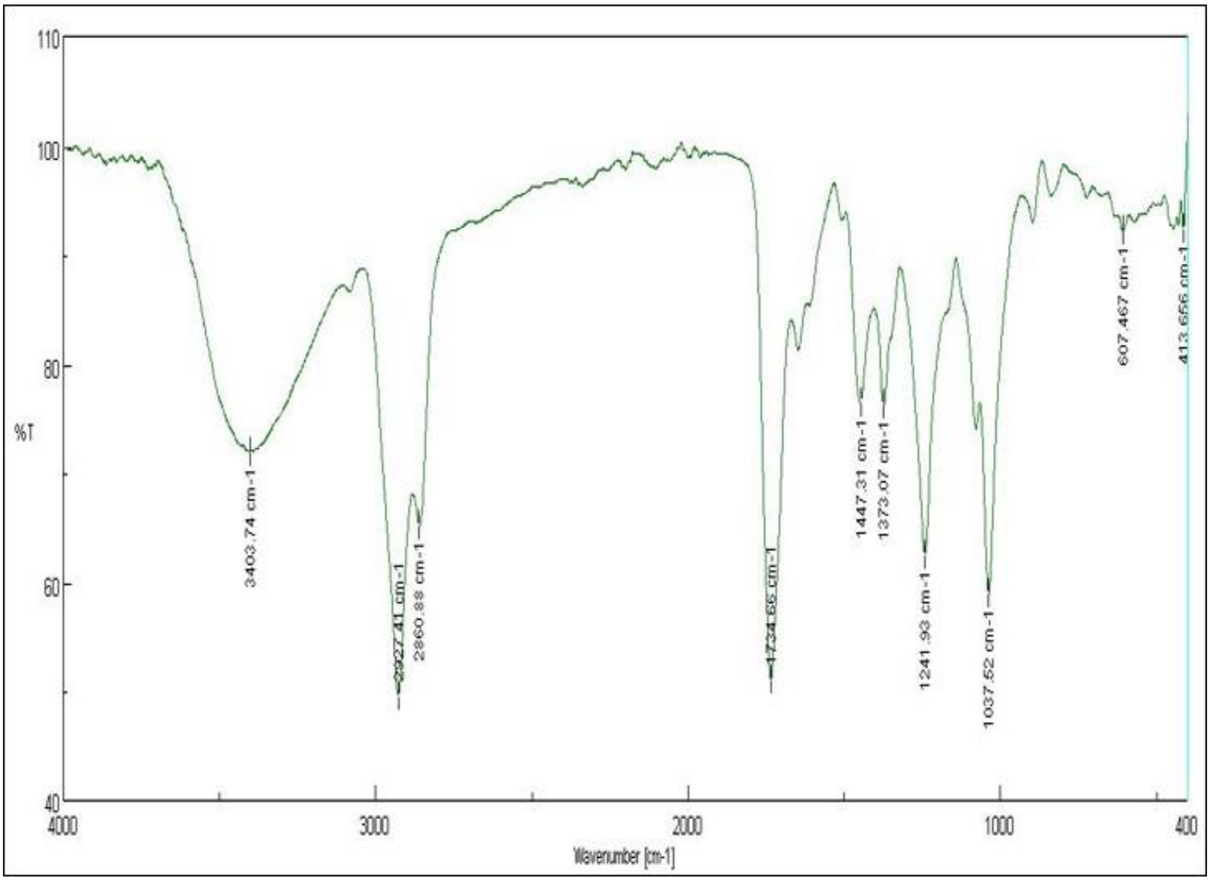

FTIR spectra of CEA extract

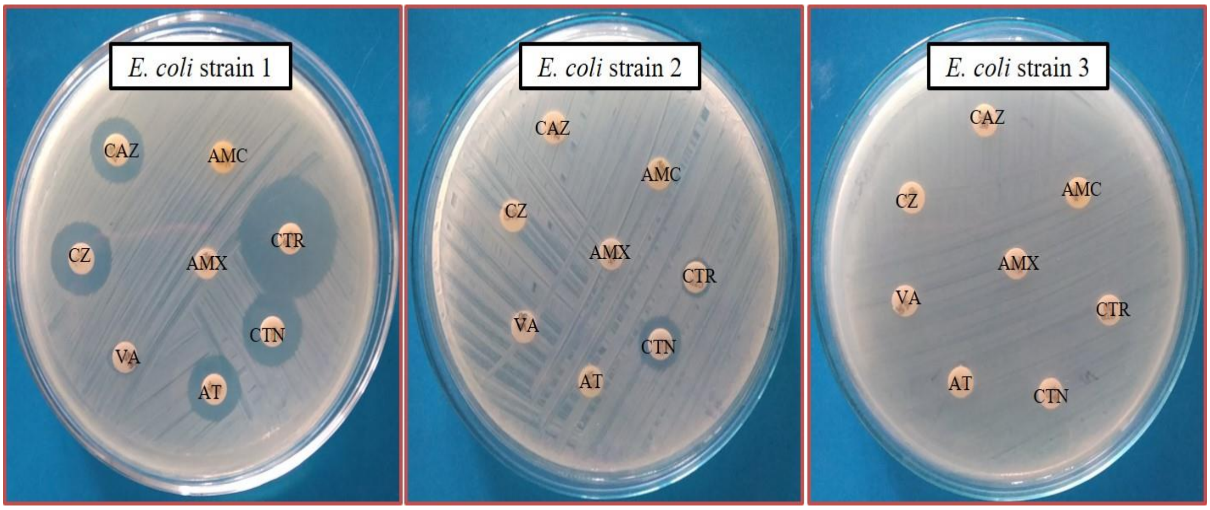

Antibiotic susceptibility of three clinical strains of *E. coli* towards different antibiotics

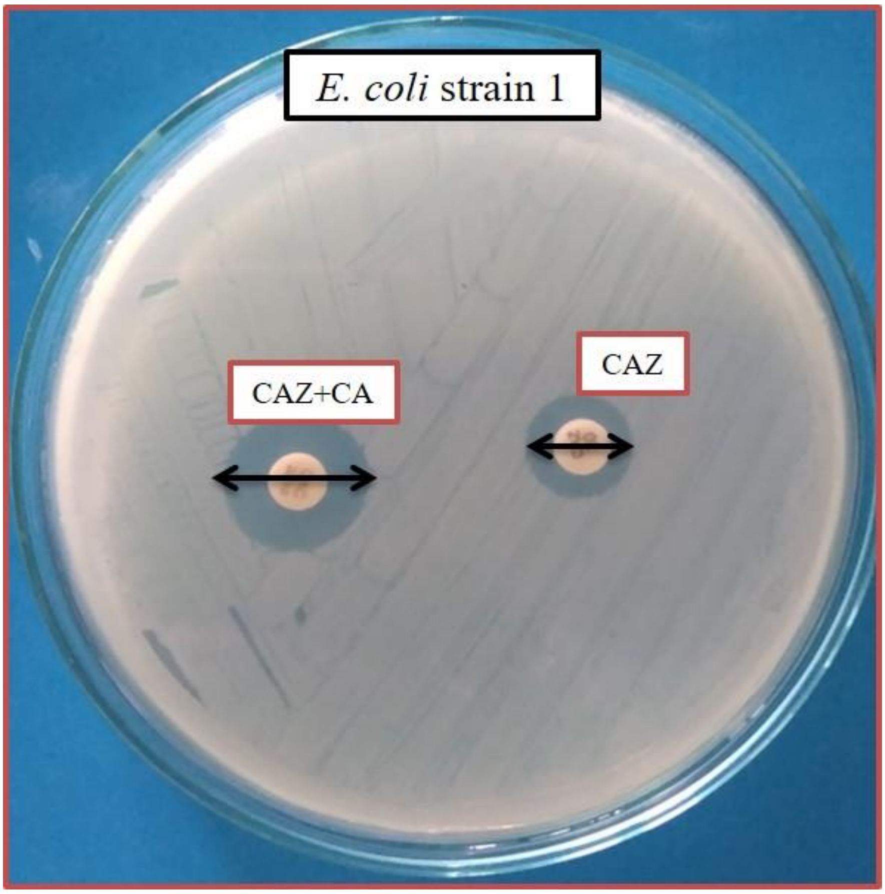

Phenotypic ESBL detection through double disk diffusion method [CAZ = Ceftazidime; CA = Clavulanic acid]
