# Supplementary material for: Efficacy of Andrographis paniculata against extended spectrum β-lactamase (ESBL) producing E. coli
Source: BMC Complement Altern Med. 2018 Sep 3;18:244. doi: 10.1186/s12906-018-2312-8 (PMC6122548; doi:10.1186/s12906-018-2312-8)
Supplement: Supplementary file 2 — Residues and bond lengths of 10 bioactive compounds of A. paniculata docked with CTX-M-15. The data in the file includes the tabular representation of the docking results showing the residues and bond lengths of 10 bioactive compounds of A. paniculata with CTX-M-15. (PDF 15 kb) [file 12906_2018_2312_MOESM2_ESM.pdf]

Table: Residues and bond lengths of 10 bioactive compounds of A. paniculata docked with CTX-M-15

**S. No**	**Compound**	**Residues**	**Bond length**
**1**	Onysilin	ASN107<br>ASN135	3.1 A^0<br>3.1 A^0
**2**	Neoandrographolide	ARG257<br>ALA273<br>ALA273<br>PRO170	3.3 A^0<br>1.9 A^0<br>3.3 A^0<br>1.9 A^0
**3**	Luteolin	ASN135<br>ASN107<br>ARG257<br>SER133	2.4 A^0<br>3.3 A^0<br>2.3 A^0<br>3 A^0
**4**	Bisandrographolide A	GLY244<br>ARG257	3.5 A^0<br>3.3 A^0
**5**	Apigenin	ASN107<br>ASN135	3.3 A^0<br>3.2 A^0
**6**	Andrographolactone	SER240	2.5 A^0
**7**	Andrographiside	ARG257<br>ASN135<br>TYR132	3.5 A^0<br>2.2 A^0<br>2.3 A^0
**8**	Andrographidine C	SER240<br>SER73<br>THR238<br>TYR108	3.4A^0, 2.8A^0, 2.5A^0<br>3.1A^0, 3.5A^0<br>2A^0, 3A^0<br>2.8A^0
**9**	Andrographidine A	ASN107<br>ARG257	3.5A^0<br>2.7A^0, 2.9A^0
**10**	Andrograpanin	ARG257	2.6 A^0
